# Brain iron content in cerebral amyloid angiopathy using quantitative susceptibility mapping

**DOI:** 10.3389/fnins.2023.1139988

**Published:** 2023-04-17

**Authors:** Breni Sharma, Andrew E. Beaudin, Emily Cox, Feryal Saad, Krista Nelles, Myrlene Gee, Richard Frayne, David G. Gobbi, Richard Camicioli, Eric E. Smith, Cheryl R. McCreary

**Affiliations:** ^1^Cumming School of Medicine, University of Calgary, Calgary, AB, Canada; ^2^Department of Clinical Neurosciences, University of Calgary, Calgary, AB, Canada; ^3^Hotchkiss Brain Institute, University of Calgary, Calgary, AB, Canada; ^4^Seaman Family MR Research Centre, University of Calgary, Calgary, AB, Canada; ^5^Department of Medicine (Neurology), University of Alberta, Edmonton, AB, Canada; ^6^Department of Radiology, University of Calgary, Calgary, AB, Canada; ^7^Calgary Image Processing and Analysis Centre, University of Calgary, Calgary, AB, Canada; ^8^Neuroscience and Mental Health Institute, University of Alberta, Edmonton, AB, Canada

**Keywords:** cerebral amyloid angiopathy, Alzheimer’s disease, iron, neuroimaging, quantitative susceptibility mapping

## Abstract

**Introduction:**

Cerebral amyloid angiopathy (CAA) is a small vessel disease that causes covert and symptomatic brain hemorrhaging. We hypothesized that persons with CAA would have increased brain iron content detectable by quantitative susceptibility mapping (QSM) on magnetic resonance imaging (MRI), and that higher iron content would be associated with worse cognition.

**Methods:**

Participants with CAA (*n* = 21), mild Alzheimer’s disease with dementia (AD-dementia; *n* = 14), and normal controls (NC; *n* = 83) underwent 3T MRI. Post-processing QSM techniques were applied to obtain susceptibility values for regions of the frontal and occipital lobe, thalamus, caudate, putamen, pallidum, and hippocampus. Linear regression was used to examine differences between groups, and associations with global cognition, controlling for multiple comparisons using the false discovery rate method.

**Results:**

No differences were found between regions of interest in CAA compared to NC. In AD, the calcarine sulcus had greater iron than NC (β = 0.99 [95% CI: 0.44, 1.53], *q* < 0.01). However, calcarine sulcus iron content was not associated with global cognition, measured by the Montreal Cognitive Assessment (*p* > 0.05 for all participants, NC, CAA, and AD).

**Discussion:**

After correcting for multiple comparisons, brain iron content, measured via QSM, was not elevated in CAA compared to NC in this exploratory study.

## Introduction

Cerebral amyloid angiopathy (CAA) is characterized by aggregation of beta-amyloid in the media and adventitia layers of small to medium sized arteries of the brain and leptomeninges (Charidimou et al., 2017). Beta-amyloid is toxic to the vessel wall, resulting in loss of smooth muscle cells and fibrosis. In late stages, there is disruption of the vessel wall with leakage of red blood cells. Consequently, CAA is a major cause of lobar intracerebral hemorrhage (ICH) and convexity sulcal subarachnoid hemorrhage (SAH). Additionally, signs of iron deposition from past asymptomatic bleeds, visible as cerebral microbleeds (CMBs) and cortical superficial siderosis (cSS), are common.

Iron accumulation in select brain regions is a feature of aging (Persson et al., 2015) and neurodegeneration, and can lead to cognitive deficits (Chen et al., 2021). Brain iron content can be estimated non-invasively on magnetic resonance imaging (MRI) using quantitative susceptibility mapping (QSM) (de Rochefort et al., 2010). Exploratory studies suggest that brain iron content and QSM signal is increased in Alzheimer’s disease (AD), a sister disease of CAA caused by beta-amyloid accumulation in the brain parenchyma in the form of senile plaques, and has been linked to declines in cognition and the transition from mild cognitive impairment to AD (Ward et al., 2014; Eskreis-Winkler et al., 2017). Additional research suggests that elevated brain iron content may particularly be linked to the onset of beta-amyloid production and aggregation by increasing activity of beta-secretase, the enzyme involved in producing beta-amyloid from amyloid precursor protein (Acosta-Cabronero et al., 2013; Ward et al., 2014; McCarthy and Kosman, 2015). However, to date there are no studies looking at brain iron content using QSM in individuals with CAA.

Given CAA displays histological evidence of iron accumulation in the occipital lobe (Bulk et al., 2018) and is associated with frequent, asymptomatic hemorrhaging, which releases iron via hemoglobin breakdown in liberated red blood cells, as well as the AD-related changes mentioned, we hypothesized that cerebral cortex iron content would be increased in CAA. We theorized that diffuse microscopic bleeding not readily apparent on MRI would alter the average tissue iron concentration, resulting in diffusely elevated QSM signal. We tested this hypothesis by comparing QSM signal between healthy controls, CAA, and AD in brain regions known to be affected by age, CAA, and AD pathology. Furthermore, we tested whether increased gray matter susceptibility was associated with cognitive impairment as whole-brain susceptibility has previously been linked to lower cognition in patients with AD (Yang et al., 2022).

## Materials and methods

### Data availability

The data that support the findings of this study are available from the corresponding author upon reasonable request.

### Participants

Participants were selected from ongoing cohort studies, Functional Assessment of Vascular Reactivity in Small Vessel Disease-II (FAVR-II; University of Calgary and University of Alberta) and the Calgary Normative Study (University of Calgary). Both studies were approved by respective institutional review boards and written informed consent was acquired.

Cerebral amyloid angiopathy and AD participants were recruited in FAVR-II. CAA participants were 60–85 years of age and were diagnosed with probable CAA according to the modified Boston Criteria v1.0 (Knudsen et al., 2001; Linn et al., 2008) after presenting with lobar ICH, transient focal neurological episodes, or mild cognitive impairment. Participants with mild AD-dementia were also 60–85 years of age and diagnosed with probable AD in accordance with the National Institute on Aging – Alzheimer’s Association (NIA-AA) core clinical criteria (McKhann et al., 2011), had Montreal Cognitive Assessment (MoCA) total score of 13–24, and were residing in the community and not a long term care home.

Normal control (NC) participants were included from both FAVR-II and the Calgary Normative Study to increase the overall sample size of this study and were recruited by community advertising. In both studies, NC were free of central nervous disorders including stroke, mild cognitive impairment, or dementia, as verified by a neurologist. NC from the Calgary Normative Study were males ≥65 years and, selected to increase the sample size and better match sex frequency and mean age compared with participants with CAA.

Participants with AD or NC were excluded if there was MRI evidence of CAA, as indicated by cSS or lobar CMBs.

### Neuroimaging

Participants underwent 3T MRI (University of Calgary: GE Signa VH/I or MR750, GE Healthcare, Waukesha, WI; University of Alberta: Siemens Prisma, Erlangen, Germany) using a 32-(University of Calgary, FAVR-II Study), 20-(University of Alberta, FAVR-II Study), or a 12-(University of Calgary, Calgary Normative Study) channel head coil. T1-weighted images were acquired using a 3D inversion-prepared fast spoiled gradient sequence (University of Calgary: TR/TE/TI = 7.8/3.2/400 ms, flip angle = 11°; 256 × 256 acquisition matrix, reconstructed voxel size = 1.0 mm × 1.0 mm × 1.0 mm; University of Alberta: TR/TE/TI = 2300/2.98/400 ms, flip angle = 9°, 256 × 256 acquisition matrix, reconstructed voxel size = 1.0 mm × 1.0 mm × 1.0 mm). A unipolar multi-echo gradient echo sequence with flow-compensation was used for QSM (University of Calgary: TR/TE = 40/4.1-36 ms; inter-echo spacing = 4.1 ms; 8 echoes; flip angle = 18°; 256 × 256 acquisition matrix; reconstructed voxel size = 0.5 mm × 0.5 mm × 2.0 mm; University of Alberta: TR/TE = 45/3.8–36.8 ms; inter-echo spacing = 4.9 ms; 7 echoes; flip angle = 17°; 256 × 190 acquisition matrix; reconstructed voxel size = 0.94 mm × 0.94 mm × 2.0 mm).

Quantitative susceptibility mapping data were processed using an in-house python implementation of previously described processing pipeline (Schweser et al., 2011; Salluzzi et al., 2017). Briefly, magnitude and phase images were calculated, a brain mask was generated from the first echo magnitude image using FSL Brain Extraction Tool (Smith, 2002), phase images were unwrapped (Abdul-Rahman et al., 2007), local magnetic field was calculated for each voxel (Smith, 2002), background field was estimated and removed via RESHARP (Sun and Wilman, 2014), and finally the magnetic susceptibility map was computed via regularized dipole inversion and deconvolution (Bilgic et al., 2014; Lee et al., 2018). Quality control checks were done to identify any maps with artifacts (i.e., motion or streaking) or evidence of ICH as these may alter susceptibility values. Maps with motion or streaking were excluded from the analysis. On the maps with evidence of ICH, brain masks were manually edited to remove the ICH region and the remaining map was retained for analysis.

To determine QSM signal in specific brain regions, the first echo of the QSM acquisition was registered to the individual’s 3D T1-weighted image using linear transformation in Freesurfer 6.0.0. The Destrieux atlas (Destrieux et al., 2010) and Aseg atlas (Fischl et al., 2002) were then transformed to the susceptibility maps using inverted transform matrices, from which average susceptibility measures of brain regions were calculated.

Brain regions of interest (ROIs) were selected based on relevance to aging and the disease groups being studied. Age-related regions included the thalamus, caudate, putamen, and pallidum (Ward et al., 2014). Frontal and occipital cortical regions were selected as they tend to be affected by CAA pathology (Charidimou et al., 2017): anterior cingulate cortex gyrus and sulcus, middle-anterior cingulate cortex gyrus and sulcus, cuneus, lingual gyrus, subcollosal area, calcarine sulcus, parieto-occipital sulcus, and suborbital sulcus. Lastly, the hippocampus was selected because it degenerates in AD (Braak et al., 1993). ROIs on the outer edge of the brain in close proximity to the skull were avoided, except for the hippocampus which has been used as an ROI in other studies, to reduce the risk of contamination by artifact or erosion during the brain mask calculation. For each ROI, susceptibility values of the left and right structures were averaged; thus, each ROI value represented the bilateral average.

### Statistical analysis

In univariate analysis, continuous data (i.e., age and MoCA score) were compared across groups using analysis of variance with Tukey–Kramer test *post-hoc* for multiple comparisons, and categorical data (i.e., sex) was compared using Fisher’s exact test.

Susceptibility values of all ROIs for all groups were standardized to the NC group of this study using the mean and standard deviation of the NC group. For example, the standardized susceptibility of the cuneus in the CAA group was calculated as: [(mean susceptibility of cuneus in CAA – susceptibility of cuneus in NC)/(standard deviation of susceptibility values of cuneus in NC)]. Positive values are consistent with greater iron concentration in the tissue. Standardized susceptibility values of cortical and subcortical regions were compared to that of the NC group using linear regression (i.e., PROC GLM in SAS) with least square means without adjustments and again while adjusting for age and sex. To account for multiple hypothesis testing, a false discovery rate-adjusted *p*-value (*q*-value) of <0.05 was applied.

To examine associations between regions with significantly greater iron content than NC and global cognition, as measured by total MoCA score, linear regression (i.e., PROC REG in SAS) was used, adjusting for age and sex. All statistical analyses were conducted using SAS version 9.4 (SAS Institute Inc., Cary, NC, USA).

## Results

### Study characteristics

Table 1 displays participant study characteristics. After exclusion of participants that did not pass quality control checks (1 NC, 8 CAA, and 2 AD), 118 remained. Of these, 83 were NC participants (37 from the Calgary Normative Study and 46 from the FAVR-II Study), 21 were CAA participants, and 14 were AD participants. Participants with CAA were older (mean 75.1 years [SD 7.6]) with 9 (42.9%) females. As expected, the NC group had higher MoCA total scores (26.2 [SD 2.3]) compared to both CAA and AD.

**TABLE 1:** Participant characteristics.

	All participants	NC	CAA	AD	*p*-Value
N	118	83^a^	21	14	−
Age, years (SD)	71.7 (6.7)	71.3 (6.3)	75.1 (7.6)	68.8 (5.9)	**0**.**01**
Female, *n* (%)	47 (39.8)	33 (39.8)	9 (42.9)	5 (35.7)	**0**.**04**
MoCA, median (IQR)	24.4 (4.4)	26.2 (2.3)	21.0 (5.9)	18.8 (3.8)	**<0**.**0001**

AD, Alzheimer’s disease; CAA, cerebral amyloid angiopathy; CNS, Calgary Normative Study; FAVR-II, Functional Assessment of Vascular Reactivity in Small Vessel Disease-II; IQR, interquartile range; NC, normal controls. Values are mean (standard deviation) or number (percentage), as appropriate. Significant *p*-values are bolded.

^a^NC group consisted of participants from the FAVR-II study (*n* = 46) and the CNS (*n* = 37).

### Group differences in magnetic susceptibility

Average bilateral raw susceptibility values (ppm) of ROIs by study group are displayed in Table 2.

**TABLE 2:** Susceptibility values of regions of interest.

	NC	CAA	AD
**Cortical regions**			
Anterior cingulate cortex gyrus and sulcus	0.004 (0.01)	0.007 (0.007)	0.013 (0.01)
Middle-anterior cingulate cortex gyrus and sulcus	−0.003 (0.01)	−0.001 (0.01)	−0.005 (0.02)
Cuneus	−0.006 (0.01)	−0.003 (0.01)	−0.002 (0.01)
Lingual gyrus	0.015 (0.0.2)	0.016 (0.01)	0.026 (0.01)
Subcollosal area	−0.026 (0.03)	−0.026 (0.02)	−0.023 (0.01)
Calcarine sulcus	0.002 (0.01)	0.007 (0.01)	0.013 (0.01)
Parieto-occipital sulcus	0.004 (0.01)	0.006 (0.01)	0.010 (0.01)
Suborbital sulcus	0.002 (0.02)	−0.002 (0.02)	0.007 (0.01)
**Subcortical regions**			
Thalamus	−0.001 (0.01)	−0.009 (0.01)	0.001 (0.01)
Caudate	0.046 (0.03)	0.045 (0.02)	0.057 (0.03)
Putamen	0.061 (0.05)	0.078 (0.03)	0.088 (0.03)
Pallidum	0.107 (0.07)	0.146 (0.08)	0.133 (0.02)
Hippocampus	−0.006 (0.01)	−0.001 (0.01)	0.001 (0.01)

Values represent average bilateral raw susceptibility values (standard deviation) of regions of interest, in ppm.

AD, Alzheimer’s disease; CAA, cerebral amyloid angiopathy; NC, normal controls.

**FIGURE 1:**
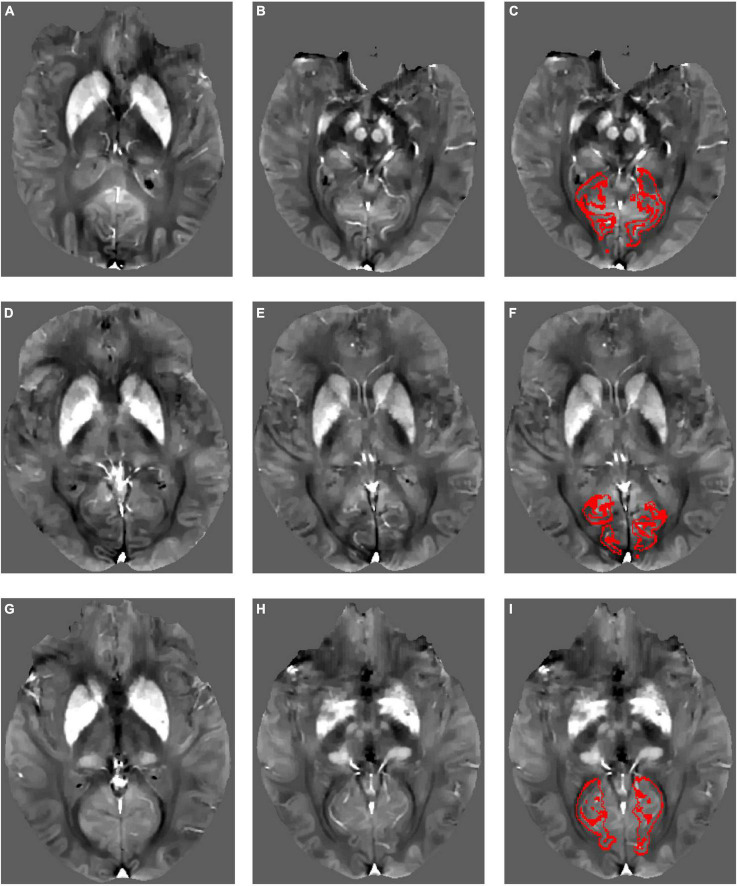
Example quantitative susceptibility maps for a participant with Alzheimer’s disease **(A–C)**, a participant with cerebral amyloid angiopathy **(D–F)**, and a normal control **(G–I)**. Normal high susceptibility is evident in the striatum in all participants, caused by age-related iron deposition. Red outlines in panels **(C,F,I)** show the calcarine sulcus region of interest within each participant’s native image space from which mean QSM values were derived.

Examples of susceptibility maps in CAA, AD, and NC are shown in Figure 1. Participants with CAA did not differ from NC on any cortical ROIs (anterior cingulate cortex gyrus and sulcus, middle-anterior cingulate cortex gyrus and sulcus, cuneus, lingual gyrus, subcollosal area, calcarine sulcus, parieto-occipital sulcus, and suborbital sulcus) or subcortical ROIs (thalamus, caudate, putamen, pallidum, and hippocampus) after adjusting for age and sex (see Table 3).

**TABLE 3:** Group differences in magnetic susceptibility across regions of interest.

	CAA				AD			
	**Unadjusted** **β (95% CI)**	***q*-Value**	**Adjusted** **β (95% CI)**	***q*-Value**	**Unadjusted** **β (95% CI)**	***q*-Value**	**Adjusted** **β (95% CI)**	***q*-Value**
**Cortical regions**								
Anterior cingulate cortex gyrus and sulcus	0.19 (−0.24, 0.62)	0.61	0.25 (−0.16, 0.67)	0.43	0.67 (0.16, 1.17)	0.06	0.65 (0.17, 1.13)	0.06
Middle-anterior cingulate cortex gyrus and sulcus	0.10 (−0.39, 0.60)	0.89	0.18 (−0.33, 0.69)	0.63	−0.13 (−0.72, 0.45)	0.70	−0.16 (−0.75, 0.43)	0.66
Cuneus	0.32 (−0.18, 0.82)	0.45	0.39 (−0.13, 0.90)	0.34	0.35 (−0.25, 0.94)	0.35	0.28 (−0.32, 0.87)	0.47
Lingual gyrus	0.06 (−0.39, 0.50)	0.94	0.06 (−0.40 0.52)	0.87	0.67 (0.14, 1.20)	0.06	0.66 (0.13, 1.20)	0.07
Subcollosal area	−0.01 (−0.45, 0.43)	0.96	0.005 (−0.45, 0.46)	0.98	0.09 (−0.43, 0.61)	0.73	0.07 (−0.46, 0.60)	0.79
Calcarine sulcus	0.51 (0.50, 0.97)	0.13	0.58 (0.11, 1.05)	0.07	**1.04** **(0.50, 1.59)**	**<0.01**	**0.99** **(0.44, 1.53)**	**0.01**
Parieto-occipital sulcus	0.14 (−0.30, 0.58)	0.76	0.20 (−0.24, 0.63)	0.61	0.43 (−0.09, 0.94)	0.23	0.40 (−0.11, 0.90)	0.27
Suborbital sulcus	−0.21 (−0.67, 0.25)	0.61	−0.17 (−0.63, 0.29)	0.63	0.31 (−0.24, 0.86)	0.35	0.33 (−0.21, 0.86)	0.37
**Subcortical regions**								
Thalamus	−0.72 (−1.23, −0.20)	0.09	−0.67 (−1.19, −0.15)	0.07	0.16 (−0.45, 0.77)	0.70	0.15 (−0.45, 0.76)	0.66
Caudate	−0.01 (−0.47, 0.45)	0.96	0.10 (−0.35, 0.55)	0.78	0.36 (−0.18, 0.90)	0.32	0.30 (−0.22, 0.82)	0.37
Putamen	0.38 (−0.06, 0.82)	0.29	0.39 (−0.05, 0.83)	0.28	0.59 (0.08, 1.11)	0.08	0.59 (0.08, 1.11)	0.08
Pallidum	0.54 (0.07, 1.02)	0.13	0.59 (0.12, 1.06)	0.07	0.36 (−0.19, 0.92)	0.32	0.35 (−0.19, 0.89)	0.37
Hippocampus	0.39 (−0.10, 0.88)	0.31	0.36 (−0.14, 0.87)	0.34	0.53 (−0.05, 1.11)	0.19	0.54 (−0.05, 1.12)	0.19

Differences between study groups and normal controls, standardized to normal control group. Positive values denote greater iron compared to normal controls in the respective regions.

Adjusted models control for age and sex. *Q* values are *p*-values corrected for the false discovery rate. Bold indicates significant difference (*q* < 0.05).

AD, Alzheimer’s disease; CAA, cerebral amyloid angiopathy.

Participants with AD had greater susceptibility than NC in the calcarine sulcus, but did not differ in the remaining cortical ROIs (anterior cingulate cortex gyrus and sulcus, middle-anterior cingulate cortex gyrus and sulcus, cuneus, lingual gyrus, subcollosal area, parieto-occipital sulcus, and suborbital sulcus) or any subcortical ROIs (thalamus, caudate, putamen, pallidum, and hippocampus; see Table 3).

### Associations with global cognition

Magnetic susceptibility of the calcarine sulcus was not associated with global cognition, as measured by MoCA total score in NC, CAA, AD, or across all participants after adjusting for age and sex, and, in the model with all participants, group (see Table 4).

**TABLE 4:** Associations between magnetic susceptibility of calcarine sulcus and MoCA total score.

All participants	NC	CAA	AD
β (95% CI)	β (95% CI)	β (95% CI)	β (95% CI)
−0.57 (−1.18, 0.05) *p* = 0.07	−0.42 (−0.87, 0.03) *p* = 0.87	−1.25 (−4.08, 1.57) *p* = 0.36	−2.69 (−6.16, 0.79) *p* = 0.12

Model with all participants was adjusted for age, sex, and group. Models separated by groups were adjusted for age and sex.

AD, Alzheimer’s disease; CAA, cerebral amyloid angiopathy; MoCA, Montreal Cognitive Assessment.

## Discussion

This study estimated iron content by measurement of magnetic susceptibility in brain regions of CAA participants using QSM. Compared to healthy controls, CAA participants demonstrated no differences in iron content in the examined regions of the frontal lobe, occipital lobe, and subcortex, including the hippocampus. However, in AD participants, increased iron was detected in the calcarine sulcus, although iron in the calcarine sulcus was not associated with global cognition across participants or in any of the study groups.

Our findings were not consistent with our hypothesis that iron content would be increased in the cerebral cortical tissue in CAA or with previous histological evidence of iron accumulation in the occipital lobe in CAA (Bulk et al., 2018) or across the brain in AD (Ayton et al., 2021).

In AD participants, we found that iron concentration was increased in the calcarine sulcus (in the occipital lobe), a region of the primary visual cortex. This finding may be linked to atrophy (which is correlated with iron accumulation (Yang et al., 2022)) of the visual cortex seen in AD (Ashok et al., 2020). Other studies of QSM in AD have had mixed findings, either finding no differences in iron content or greater iron when compared to controls in the occipital lobe (Ramos et al., 2014; Damulina et al., 2020; Rao et al., 2022). Overall, studies found increased iron in subcortical regions (globus pallidus, caudate, putamen, and hippocampus) when compared to healthy controls but comparisons of cortical regions were inconsistent (Li et al., 2020; Uchida et al., 2022).

Limitations of this study include the small sample size, particularly in the CAA and AD groups. We cannot exclude the possibility that a larger study would find differences between CAA and NC, although the differences would probably be small. Regarding the QSM methodology, regularization of the processing pipeline has yet to be optimized, which may introduce bias into the results and contribute to the lack of significant findings. This may be alleviated by ultimately achieving consensus on a standardized QSM post-processing pipeline. While AD participants with MRI markers indicative of comorbid CAA (e.g., cSS, lobar CMBs) were excluded, other MRI markers of cerebral small vessel disease (CSVD), such as white matter hyperintensities, were present to varying degrees in the AD participants. This may lead to difficulty differentiating between AD and CSVD and the possibility that our findings in the AD group may be partly reflective of coexisting CSVD. The inclusion of multiple head coils due to different sites may have introduced bias in our findings (Panman et al., 2019). The use of large ROIs may have obscured smaller regional differences. Finally, adjusting the *p*-values for multiple comparisons reduced the statistical power.

This study examined brain iron content in CAA using QSM. Although we did not find evidence of increased average tissue iron content in CAA, contrary to our original hypothesis, we did find that iron content was higher in the brain in AD. However, QSM may still ultimately play a role in CAA assessment because it delineates microbleeds and cortical superficial siderosis with better contrast and less blooming artifact than conventional susceptibility-weighted imaging. Future larger studies are needed to determine whether QSM-defined hemorrhagic lesions are better diagnostic and prognostic markers of CAA than those identified on conventional susceptibility-weighted imaging.

## Data availability statement

The raw data supporting the conclusions of this article will be made available by the authors, without undue reservation.

## Ethics statement

The studies involving human participants were reviewed and approved by Conjoint Health Research Ethics Board, University of Calgary. The patients/participants provided their written informed consent to participate in this study.

## Author contributions

AB, EC, KN, and MG contributed to the data collection. DG contributed to the development of quantitative susceptibility mapping processing pipeline. CM contributed to the processing of data. BS and CM contributed to the quality control. AB, BS, CM, ES, and FS contributed to the data analysis. BS, CM, ES, and RC contributed to the interpretation of results. BS, CM, and ES contributed to the writing of manuscript. All authors contributed to manuscript revision, read, and approved the submitted version.
